# “We can tell a good teacher who cares, understands, and can be confidential about it”: youth and caregiver experiences with HIV disclosure to schools in Kenya

**DOI:** 10.3389/fpubh.2023.1172431

**Published:** 2023-07-25

**Authors:** Hellen Moraa, Irene Njuguna, Cyrus Mugo, Anne Mbwayo, Florence Nyapara, Calvins Aballa, Anjuli Dawn Wagner, Dalton Wamalwa, Grace John-Stewart, Irene Inwani, Gabrielle O'Malley

**Affiliations:** ^1^Kenyatta National Hospital, Research and Programs, Nairobi, Kenya; ^2^Departments of Global Health, University of Washington, Seattle, WA, United States; ^3^Department of Psychiatry, University of Nairobi, Nairobi, Kenya; ^4^Department of Pediatrics and Child Health University of Nairobi, Nairobi, Kenya; ^5^Department of Epidemiology, University of Washington, Seattle, WA, United States; ^6^Department of Medicine, University of Washington, Seattle, WA, United States; ^7^Department of Pediatrics, University of Washington, Seattle, WA, United States

**Keywords:** youth living with HIV, disclosure, school, caregiver, barrier

## Abstract

**Introduction:**

Disclosure of one's HIV status to others is often difficult due to the fear of stigma. However, disclosure may facilitate receiving social support. Many youth living with HIV (YLH) are enrolled in school as better treatments have improved the health and survival of children with HIV. There is no structured process for disclosure at school for YLH and their caregivers. We sought to understand school disclosure experiences among YLH and their caregivers and assess the need for the development of a structured disclosure intervention tailored to school settings.

**Methods:**

We conducted in-depth qualitative interviews with 28 school-going YLH aged 14–19 years and 24 caregivers of YLH. Interviews were conducted in English and Swahili, transcribed, and translated. The transcripts were uploaded to Atlas.ti 9 for thematic analysis.

**Results:**

YLH and caregivers clearly articulated the benefits of disclosing to school staff. Disclosure to school staff was seen as the first step to receiving support for medication storage, adherence, and clinic attendance. However, disclosure was also perceived to be a very complicated and stressful process. Fear of stigma drove caregivers and YLH toward careful planning of when and to whom to disclose. Distrust of school staff was a significant barrier to disclosure, even among those who clearly articulated the benefits of disclosure. Disclosure to school staff largely resulted in positive experiences; the immediate reactions were positive or somewhat neutral and confidentiality was upheld. The anticipated benefits of practical and emotional support were demonstrated by the school staff to whom the HIV information was disclosed.

**Conclusion:**

Disclosure of HIV status to someone at school is necessary to receive support for medication adherence. Stigma and the lack of structured support for the disclosure process at school often hinder YLH and their caregivers from disclosing. YLH would benefit from better support at schools, including policies to facilitate disclosure that address the caregiver and YLH's fear of stigma and loss of confidentiality. School policies could also provide guidance on whom to disclose to and available post-disclosure support.

## Introduction

Youth living with HIV (YLH) have poorer health outcomes than adults and may benefit from additional psychosocial support (1). Poor medication adherence, loss to follow-up, and mortality are common challenges among YLH receiving HIV care in high-burden countries in sub-Saharan Africa (SSA). Youth HIV disclosure to others has benefits and could facilitate support for adherence, emotional health, and overall HIV care (2–4). However, disclosure is often challenging; frequently, there is fear that self-disclosure may result in negative outcomes such as shame and stigma (5–7). As a result, disclosure is often limited to a few trusted people, even within the family setting (8). YLH self-disclosure is based on trust and may vary across settings. In Kenya, a study found that youth older than 18 years were more likely to self-disclose to family members than to non-family members (1). Prior studies also suggest that YLH self-disclosure to peers and sexual partners including those who are married is low (1, 2). A systematic review of studies conducted in SSA found that disclosure challenges were an important issue for YLH and support within the school setting was inconsistent (3, 4).

As HIV treatment has improved over time, morbidity and mortality have decreased for children and adolescents and the majority of YLH are enrolled in schools. Disclosure to school staff or peers has the potential to increase support to YLH for adherence to medication and clinic visits. However, few YLH disclose their HIV status to anyone in their school community, often resulting in a lack of support for medication storage, adherence, and clinic attendance (9, 10). Often, YLH are conditioned to maintain the secrecy of their HIV status to avoid negative responses such as rejection (4, 5).

For YLH in boarding schools—which is common in sub-Saharan Africa, especially for secondary education—disclosure is particularly critical to obtain support for medication storage, reminders, and psychosocial support. For those in day school, storage for medications and medication reminders can happen at home. Obtaining permission to miss classes in order to attend a clinic during school hours is challenging without disclosure in both boarding and day schools. In addition, YLH could greatly benefit from psychosocial support and gain a sense of acceptance within the school setting. It is also during this phase of development that youth are developing meaningful relationships with peers and there could be fear that disclosure of their HIV status may jeopardize this. Careful and structured disclosure to staff and peers at school is therefore critical to obtaining support for medication adherence, clinic visits, and psychosocial support (9, 11).

The data on how YLH and caregivers navigate HIV disclosure in the school setting are scarce. A better understanding of caregiver and YLH disclosure experiences within the school environment would provide relevant information that could support the development of interventions aimed at improving HIV disclosure at school. This study sought to understand caregiver and YLH experiences and perceptions of HIV disclosure in the school environment using in-depth interviews.

## Methods

This qualitative study was conducted between March and October 2021 at 12 HIV clinics across three counties (Nairobi, Kajiado, and Homabay) in Kenya with an adult HIV prevalence ranging between 4% and 21% (12). Eligible participants were recruited by study staff during their regular clinic appointments. YLH were eligible if they were aged 14–19 years, aware of their HIV status (confirmed by the caregiver and healthcare worker), had ever been on antiretroviral therapy (ART) while in school, and provided informed consent/assent. Caregivers were eligible if they were the primary caregiver for YLH, aged ≥18 years, and provided informed consent. YLH in both boarding and day schools were included. Caregivers and youth aged 18 years provided informed consent.

We interviewed 24 caregivers and 28 YLH. Among the caregivers interviewed, 3(12%) were men and the median age (IQR) was 44 (38, 49). The majority, 21(88%), were a parent to YLH and (9) 38% had YLH who had been to boarding school (Table 1). Of the YLH who participated in this study, 13 (46%) were boys and their median age (IQR) was 17 (13, 14). There were 10(36%) YLH enrolled in primary school and those in boarding school were 11 (39%). More than half (64%) of their caregivers were living with HIV and those paired with their caregivers for this interview were 13 (46%). Only a few (18%) YLH had been on ART for <5 years (Table 2).

**Table 1:** Characteristics of caregivers who participated in IDIs.

**Characteristics**	***N* = 24 *n* (%), median (IQR)**
Age	44 (38, 49)
Male	3 (12%)
Received post-primary education	13 (54%)
Parent of a YLH	21 (88%)
HIV positive	19 (79%)
On ART	19 (100%)
Has YLH who has been to boarding school	9 (38%)

**Table 2:** Characteristics of YLH who participated in in-depth interviews.

**Participant characteristics**	***N* = 28 *n* (%), median (IQR)**
Age	17 (16, 18)
Male	13 (46%)
**Schooling**	
Primary school	10 (36%)
Secondary school	18 (64%)
Reported history of being in boarding school	16 (57%)
In boarding school	11 (39%)
On ART	28 (100%)
**ART duration**	
Less than 5 years	5 (18%)
More than 5 years	23 (82%)
Parent is primary caregiver	21 (75%)
Caregiver is living with HIV	18 (64%)
Paired with caregiver	13 (46%)

We purposively sampled YLH across 4 different categories: YLH in boarding school (8 IDIs), those in day school (8 IDIs), those transitioning to a new school in the next academic year (8 IDIs), and those who had changed schools in the past year (4 IDIs). These categories provided a balanced representation of youth in different school settings as well as views on considerations made during the transition to different school settings. We used semi-structured interview guides that were pilot-tested and refined. Interviews focused on YLH's experiences with medication use and storage, obtaining permissions for clinic attendance, experiences/perspectives on disclosure in school, stigma in the school environment, factors influencing school choice, and strategies to improve YLH experience in school. There were five main questions in the youth interview guide and seven questions in the caregiver interview guide. Each guide had several predetermined probes (Table 3). Interviews were conducted by an experienced qualitative researcher (HM) either by phone (at home) or in person (in the clinic) in English or Kiswahili depending on YLH/caregiver preference. Interviews lasted 20–45 min and were audio-recorded, translated as needed, and transcribed verbatim. Saturation was determined when no new themes emerged based on the researchers' (HM and INN) review of the interviews and debrief summaries.

**Table 3:** Interview guides.

**Semi-structured interview questions for adolescents/youth**	
Question 1	When Do you, or other adolescents who are living with HIV, are at school and feel sick, what do you/they do? • Where do you go for care? Who do you tell and what do they usually do? ° Do you attend a different clinic than your regular HIV clinic to get your care? Why? ° What is the process to ask for permission to go to clinic? ■ What information does the school need to give permission? ■ How long do you have to wait to get permission? ■ What if you have exams? How is what you do when you are at school different than what you might do if you felt sick and you are at home? • How is it the same? • Could you give an example of a time you needed medical care in school and got good support? What was done? How did that make you feel? • Could you give an example of a time you needed medical care in school and got poor support? What was done? How did that make you feel? • What could the school do to make the experience of attending clinic while in school better?
Question 2	What is it like taking medicine when you are in school? **ASK for ALL** ° How does taking medicine fit into your school schedule? ° Have you experienced medicine side effects when in school? What did you do about it? **ASK for BOARDING ONLY** ° How do you store your medicine when in school? ■ Is this ok with you? Why? ° How do you take your medicine when in school? ■ Is this ok with you? Why? ■ Does the school provide you clean water and food to support you to take your medicine? ° Who, if anyone, supports you to take your medicine when in school? ° Have you noticed other students who take medicine in school?
Question 3	Do you attend a support group? ° Do you think the support group helps you? How? Does the support group help you with managing your HIV while you are at school? Why or why not? ° Would you be interested in a support group that helps you manage your HIV when you are in school? Would you like this to be separate from your regular support group (if any)? Why or why not? ° How would you like the support group look like? • Prompts: ehealth support group (WhatsApp, text messages), general health talks in school on various diseases, health clubs for adolescents with any chronic illness,
Question 4	Have you told anyone that you have HIV? If so, why did you decide to tell that person? How did they react? ° Have you told anyone in school you have HIV? If yes, why did you decide to tell that person? How did they react? ° Do you think HIV-positive students should disclose their HIV status to someone at school? Why or why not? ° What do you think schools can do to help you and other students feel more comfortable talking about HIV?
Question 5	As you know, when it comes to time for upper primary or secondary school, some students go to day schools and some students to boarding school. ° Were you involved in the decision to choose boarding/day school? If yes, why did you choose day/boarding school? If not, who made the decision? ° Were you concerned that it might be harder to manage your clinic visits and medicine if you were at a boarding school? How do you think medication storage when in school could influence your decision to attend boarding or day schools? Why? ° How does your experience receiving medical care while in school influence what schools you attend? Do they make you want to go to boarding school or day school? Why? ° How do you think what other people think about HIV in school would influence your decision to attend boarding or day school? Why?
**Semi-structured interview questions for caregivers**	
Question 1	What kind of things do you talk about to your teenager regarding school? *Prompts: How to stay healthy, sexual education, maintaining good grades* •Where do you get the information you share with your teenager? ° Prompts: Lived experience, media, other mothers, church, support groups •What kind of support do you think caregivers of teenagers need to talk to their teenagers? ° Prompts: parenting classes, parenting material, support groups. Why •What other topics do you talk about only to your teenager who is living with HIV? ° Prompts: Medication use, what to do when unwell, HIV disclosure, stigma. •Where do you get information to talk to your teenager who is living with HIV? ° Prompts: Lived experience, media, other mothers, church, support groups What kind of support do you think caregivers of teenagers living with HIV need to talk to their teenagers? ° Prompts: parenting classes, parenting material, support groups. Why? •Could you give an example of a time a talk with your teenager went well? What material did you use as you talked to them? Did you feel you needed more support? •Could you give an example of a time a talk with your teenager did not go well? What material did you use as you talked to them? Did you feel you needed more support?
Question 2	When your teenager is in school, what do they do if they are unwell? Who supports your teenager when he/she is unwell in school? •Could you give an example of a time your teenager told you they had a good experience in school when they were unwell? •Could you give an example of a time your teenager told you they had a bad experience in school when they were unwell? •How can the school improve this process to make it better for your teenager?
Question 3	When your teenager is in school, do they ever have to attend the HIV clinic? •What processes or actions do you as a caregiver have to do with the school or someone at the school to make sure your teenager attends clinic? •How do you feel this process or actions are working for your teenager? •What could the school do to make this process or actions better?
Question 4	What has your teenager told you about taking medicines when in school? ASK FOR BOARDING ONLY •How does the school require medicine to be stored and used? •What do you think about this school requirement? •Does the school provide clean water and food to support your teenager to take medicine? •Could you give an example of a time your teenager talked to you about a good experience taking medicine in school? •Could you give an example of a time your teenager talked to you about a bad experience taking medicine in school? ASK FOR ALL • What kind of support do you think your teenager and other teenagers living with HIV need to take medicine when in day school? What about boarding school? Why or why not? ° Prompts for day school ■ Have selected teachers to talk about medication experiences as needed, allow them to come a little late or leave school early ° Prompts for boarding school ■ Someone to support them. Who would this person be? ■ Flexibility in where to store medicine. What options would work? ■ Reminders: alarm clocks ■ Alternative packaging: medicine boxes
Question 5	Do you attend a support group? •Does the support group help caregivers of teenagers living with HIV on school issues? How? •Would you be interested in a support group of caregivers of teenagers living with HIV to support school-related issues? Why or why not? ° Would you like this to be separate from your regular group? Why or why not?
Question 6	Have you disclosed your teenager's HIV status to anyone in school? If so, why did you decide to tell this person? How did they react? •Did you talk to your teenager before disclosing their HIV status? What did your teenager think? How did they react? •Do you think caregivers of teenagers should disclose the teenager's HIV status to someone in school? Why or why not? •Apart from disclosing your teenager's HIV status, did you talk to anyone at the school about your teenager's need to take medicine or to miss class sessions to attend clinic? If yes, how did that go?
Question 7	As you know, when it comes to time for upper primary or secondary school, some students go to day schools and some students to boarding school. •Who made the decision for your teenager to go to boarding or day school? If you did, why did you choose boarding or day school? If you did not, who made the decision? •Were you concerned that your teenager would stop taking their medicine if they were in boarding school? Why? Why not? •Were you concerned that your teenager may be discriminated because of their HIV status? Why? Why not?

The transcripts were then uploaded to Atlas.ti 9 for coding and thematic analysis (15). Two coders (INN and HM) then developed a codebook through an iterative process. Initially, each coder read four transcripts and developed initial codes and their definitions. The codebook was further refined and tested on additional transcripts. Once the final codebook was agreed upon, each coder did coding for half of the transcripts (primary coding) followed by a review of each other's coded transcripts (secondary coding). Disagreements were resolved through discussion and consensus.

The study received ethical approval from the Kenyatta National Hospital Ethical Review Committee (Ref: P320/06/2020). Approval was also obtained from county administrators and clinic leadership.

## Results

The findings from this study were categorized into four themes: (1) caregivers and YLH recognize that disclosure at school is beneficial; (2) disclosure is often difficult and stressful mainly as a result of fear of stigma; (3) mistrust of school staff was often a barrier to disclosure; and (4) disclosure often resulted in positive post-disclosure experiences.

### Caregivers and YLH recognize the benefits of HIV status disclosure at school

Both caregivers and youth believed better support for HIV care could follow disclosure at school. For example, they pointed out how disclosure to school staff was important for general support medication adherence.

“*I think this [disclosure to school staff] is something that even parents understand, and once you inform the caretaker [at school] who is an adult, she will take the responsibility of reminding them. I was advised by another parent to be free with the caregiver [at school] and disclose to her so that she can be reminding my daughter to take her medication.”* (*Mother of a 16-year-old girl who switched from boarding to day school)*

“*I think if I will be able to ask one of the teachers to help in keeping drugs to be able to remind me when it's time to take them [I] will be okay*.” (*15-year-old girl transitioning to secondary school)*

“*We can tell a good teacher who cares, understands and can be confidential about it and he/she can even be talking to the students.” (Mother of YLH in day school)*

“*When I was keeping medicine for myself, I used to have a hard time to go take them because other students used to be all over, that's why I opted to disclose to this teacher in order to be keeping drugs for me.” (17-year-old boy in boarding school)*

Often, caregivers and YLH reported that support for medication and clinic appointments was the main motivation for disclosure.

“*… need for permission to go to the hospital is what made me to disclose.” (15-year-old girl in day school)*

“*I think it would be better for the parents and the teachers, and if they have a dispensary with a nurse, to disclose and discuss so that the teachers will also be involved in their taking ARVs.” (Uncle of a 16-year-old boy in day school)*

Based on their belief in disclosure's potential benefits, some of the caregivers discussed the perceived benefits of discussing disclosure at school with their YLH. This was especially notable for those planning to attend boarding school. However, disclosure was still thought to be important for youth attending day school as this made it easier to get permission if the youth ever needed to visit a clinic during school hours.

“*He had wanted a boarding school and so we had earlier talked, and I told him disclosing will help him a lot, and the teachers I am going to hand him over to will care for him the same way I do at home.”* (*Mother of YLH in boarding school)*

*Disclosing to the teacher is good because they spend a lot of time with the students during the day over weekdays, we only have them over the weekend. … It [disclosure] is good because even when they [YLH] need to attend clinic to go for their medication you just ask the teacher for permission.”* (*Mother of a 15-year-old girl in day school)*

### Disclosure at school is complicated/stressful

The decision to disclose YLH HIV status to school staff or peers varied across the participants interviewed. Overall, the decision to disclose was difficult, largely because of fear of stigma and discrimination toward YLH. Some caregivers and YLH outrightly decided they would not disclose, while others decided they would disclose only if they had a well-thought-out plan on how to go about it.

“*For school, it [disclosure] is a no, because you never know one of them might leak the information about those who are positive and make it go viral.”* (*16-year-old boy in day school)*

“***Interviewer:*** *Do you think young people like you living with HIV and in school should disclose to someone at the school?* ***Respondent:*** *No, the biggest fear is people knowing I have HIV/AIDS is discrimination” (16-year-old boy, day school)*

“… *there are teachers of different backgrounds who may not be able to understand students with HIV and may discriminate against them due to their status. Before a parent discloses, they need to be extremely sure of who they are talking to and not to disclose to just anyone*.” (*Mother of a 15-year-old girl transitioning to secondary school)*

Caregivers who were considering disclosing at school shared experiences of how they had planned on disclosing their adolescent's HIV status.

“*When he was done with his primary level education, we sat down and I told him that I would accompany him on his day one of admission in secondary school and talk to the school matron to be helping to keep the drugs because it's not good when he keeps the drugs in the student school box due to the frequent inspection that is always conducted in schools that might expose him to other students, hence stigmatization.”* (*Mother of YLH in boarding school)*

“*I also told her that when we would go to school, I had to tell the school principal about her status and we all agreed on that*.” (*Mother of a 19-year-old girl in boarding school)*

*We can disclose to only one person and that person can be reminding them and make sure they take their medication … only the matron and she should be advised to keep that information confidential because this is very sensitive information which if the rest get to know, it can really discourage the student, and some may even stop taking the medication. (Mother of a 14-year-old girl transitioning)*

Some of those who decided to disclose to school staff felt that they did it out of obligation.

“*I felt bad [about disclosing my HIV status to the matron] but didn't have an option because all that I needed was education.”* (*18-year-old girl in day school)*

On some occasions, YLH reported negative or unsupportive reactions from those to whom they disclosed their HIV status at school. Some of these scenarios pointed to school staff who were unsure of what to do upon hearing the disclosure and the fact that schools may lack a clear structure on how to support YLH.

“*The nurse got informed by my parents [about my HIV status] on the admission day … so that she [could] keep medicine for me….Based on her facial expression she looked worried.” (19-year-old boy in boarding school.)*

### Mistrust of school staff is a barrier to disclosure

Although caregivers and YLH believed that there could be benefits associated with disclosure at school, this did not always translate into willingness to disclose to school staff. There was a constant struggle between the belief that disclosure would result in the much-needed support for medication adherence (especially in boarding school) and actual disclosure. This was evident among caregivers, who, despite articulating the benefits of disclosure, did not disclose to school staff or while they disclosed and got good support themselves, did not advocate for disclosure for others.

***Respondent*** *: “The only person who knows [the youth HIV status] is her aunt who also happens to be her teacher in school, so she is the one who helps her to keep the medicine as well.”*

***Interviewer:*** “*Do you think parents to the adolescents should disclose their status to anyone in school?”*

***Respondent:*** “*No, they should not (disclose).”*

***Interviewer: “*** *What of those their children attend boarding schools, should they disclose to anyone in school?”*

***Respondent*** *: “No.” (Mother of YLH in day school)*

**“** *The issue is finding someone to trust because you can disclose, and it ends up harming the student.” (Mother of YLH in boarding school)*

This was evident among YLH who were unsure of some teachers' ability to maintain confidentiality

“*There are some teachers if you disclose to them some things it turns to be the topic of discussion in staff room (19-year-old girl in boarding school)”*

For YLH, trust was a necessary prerequisite to disclosure at school. YLH noted that while it may seem easier to disclose to a teacher than a fellow student, one had to still carefully choose the kind of teacher to disclose to. Youth identified easily with teachers who seemed friendly and trustworthy enough to disclose to.

***Interviewer*** *: “Why did you disclose to the teacher?”*

***Response*** *: “Teachers can keep secret unlike fellow students who can turn their back against you anytime and walk around to gossip.”*

***Interviewer*** *: “What made you to disclose to that teacher?”*

***Response*** *: “It's how he was talking to me.” (15-year-old girl transitioning to secondary school)*

When mistrust could not be overcome, caregivers/YLH sometimes used illness substitution to obtain support for HIV care. Some caregivers reported the YLH had another common illness such as tuberculosis or asthma that required regular visits to clinics and medication use.

“*I talked to his teacher and explained to him that my child is suffering from TB [tuberculosis] and … always [has] a routine checkup, so in the event that he gets sick he can reach me on my phone so that I can come over and take him to the hospital since the school is only 5 min' walk from home. I never explained so much to him (teacher).” (Mother of YLH in boarding school)*

### Disclosure to school staff resulted in positive post-disclosure experiences

Caregivers/YLH who did disclose portrayed varied disclosure scenarios, such as caregiver-initiated disclosure with or without youth permission, or caregiver and youth agreeing on the need to disclose, and in some cases, youth disclosing without caregiver permission.

“*I'm the one who disclosed to her [the teacher] even though she was shocked by the information, but I made up my mind to let at least one of the teachers know … It was the feeling of perhaps whenever I have a challenge one can always come to my rescue*.” (*16-year-old girl who switched from boarding to day school)*

“*My mother is the one who disclosed to the school matron on the admission day and then the matron took the drugs to the deputy office for easy access.”* (*16-year-old girl in boarding school)*

On actual disclosure, YLH and caregivers reported that the reactions were positive or mostly neutral and often led to the anticipated support.

***Interviewer*** *: “How did the person you disclosed to react?”*

***Response*** *: “He offered support by asking me to be diligent taking my medicine. (19-year-old girl in day school)”*

“*For the principal, he seemed understanding and concerned, he also was very encouraging by telling us that this is life, and everyone has a purpose in life as long as one knows what brings him to school. He encouraged me and I felt comfortable”* (*Mother of a 17-year-old in boarding school*

“*The reaction was not bad because she was not the only one, there were many who are on medication and under the care of the same school staff and in the knowledge of the head teacher. I think this is something that even parents understand, and once you inform the caretaker who is an adult, she will take the responsibility of reminding them. I was advised by another parent to be free with the caretaker and disclose to her so that she can be reminding my daughter to take her medication.”* (*Mother of a 16-year-old who switched from boarding to day school*

“***Respondent:*** *I was admitted late in form one and so the deputy was a bit curious as to why that happened, that's when my mother disclosed to her*. ***Interviewer:*** *How did he react?* ***Respondent:*** *He reacted normally and even went ahead to tell me that there are others (students), so I should not panic.” (19-year-old girl, boarding school*)

Sometimes, disclosure to someone at school resulted in the loss of confidentiality. One such incident was reported by a participant in boarding school and when talking about when giving recommendations on how taking medication in school can be improved, she noted that the person to whom the information is disclosed to needs to maintain confidentiality.

“*School managers should be careful with whatever they say at a particular point and not to try to embarrass the affected students when under influence of alcohol because there is a lot of trust that parents and students accord them and should try to keep it confidential*.” (*18-year-old girl who switched from day to boarding school*

Additional quotes aligned to each of the four themes are included in Supplementary Table S1.

## Discussion

Caregivers and YLH who participated in this study highlighted the significant advantages of disclosure at school while underscoring that the process is challenging. Disclosure to school staff was reported as an important step to ensure adherence to medication, psychosocial support, and permission to attend a clinic during school time. However, disclosure was also mired in fear of stigmatization. Caregivers and youth did not trust that school staff or peers would keep their HIV status confidential nor necessarily respond to the disclosure in a supportive manner. However, those who did disclose their HIV status to school staff largely had a positive outcome and received the support they needed for successful care.

Adolescence is a period of rapid physical, cognitive, and psychosocial growth and is associated with many challenges (16). Evidence points to the need for strategies focused on improving youth health during this period as they transition to adulthood and through school transitions (17). Structural factors such as access to education, and environmental factors such as safe and supportive families and schools are important during this period (13). Young people love to have a sense of belonging within a family, among peers, and in a supportive community environment to enable them to stay healthy (18). This is especially critical for YLH who have additional challenges with stigma, parental loss, and managing a chronic illness.

As mentioned in our study, other studies have reported that HIV disclosure is difficult due to fear that others' knowledge of one's status will result in stigma and discrimination (3, 14, 19). However, our study focuses on disclosure barriers specific to school-going YLH. In both boarding and day schools, there are expectations of conformity to standard schedules, which may negatively impact YLH's ability to adhere to medication or clinic visits. Although school staff could potentially facilitate the navigation of these institutional barriers, YLH and caregiver mistrust of school staff was common in our study, and other coping mechanisms such as substituting illness were used to either get permission to attend the clinic or explain daily medication use to peers, similar to what was found in Uganda (7, 18). Selective disclosure within the family and to peers may be easier as it is based on trust built over time whereas disclosure to teachers/school staff may be more challenging as they are seen as a source of authority and are often unfamiliar at the outset (3, 14, 19–21).

Although theoretically, schools may be an ideal venue to offer support to YLH, they often lack structured support systems for disclosure of HIV status and medication adherence (10, 16, 22, 23). For example, providing detailed and correct information about HIV has been shown to reduce stigma in community settings and could be adapted for school settings (24, 25). In a recent boarding school-based intervention (Red Carpet Program) implemented in some boarding schools in Kenya, school-wide HIV education followed by disclosure education for focal persons on the school health committee or who serve as Adolescent Health advocates resulted in better linkage to care (26). Subsequently, YLH who were supported to disclose to school staff were offered treatment literacy education, counseling, medication storage, and easy access as well as support for clinic visits (26). The development of close partnerships between schools and stakeholders who are already providing support to YLH such as health facility staff at HIV care clinics is useful (22).

In summary, disclosure of HIV status at school is key to receiving medication adherence support but is often hampered by stigma, which negatively impacts YLH clinical outcomes. Schools can support the disclosure process through policies that address caregiver and YLH fears and support decision-making. They can also provide guidance at school entry on whom to disclose to and about available post-disclosure support. Positive experiences with disclosure can be leveraged to encourage more YLH and caregivers to disclose.

## Data availability statement

The raw data supporting the conclusions of this article will be made available by the authors, without undue reservation.

## Ethics statement

The studies involving human participants were reviewed and approved by Kenyatta National Hospital/University of Nairobi Ethics and Research Committee. Written informed consent to participate in this study was provided by the participants' legal guardian/next of kin.

## Author contributions

IN obtained study funding and served as the principal investigator. HM conducted interviews. IN, HM, and GO'M developed the codebook and conducted the analysis. IN and HM wrote the first draft of the manuscript. CM, AM, FN, CA, AW, GJ-S, and II reviewed the manuscript. All authors contributed to the article and approved the submitted version.
