# Supplementary material for: “We can tell a good teacher who cares, understands, and can be confidential about it”: youth and caregiver experiences with HIV disclosure to schools in Kenya
Source: Front Public Health. 2023 Jul 25;11:1172431. doi: 10.3389/fpubh.2023.1172431 (PMC10407401; doi:10.3389/fpubh.2023.1172431)
Supplement: Supplementary file 1 [file Table_1.DOCX]

| **TIMIZA Study** |
| --- |
| **Adolescent**  **In-Depth Interview Guide** |

**Interviewer Instructions:** Copies of the informed consent form should be provided to the participant and read aloud for the benefit of those who cannot read. Assent will be obtained from participants aged 14-17 with consent from parents and informed consent will be obtained from participants aged 19. Participants should be provided an opportunity to ask any questions. Verbal agreement to participate should be taped.

The following set of questions is a guide. Try to ask all the questions below in the order given, but it is more important to maintain the flow of discussion. Suggested probes have been included.

**Welcome and Introductions**

**Before turning on the recorder, start with the following introductory script:**

*Hi, my name is ____________. Thank you for agreeing to talk to me today. As mentioned earlier, I am a researcher and I am trying to learn more about how schools support HIV care for teenagers and young adults. Doctors and researchers have special knowledge about health and illness, but you are the expert on what it is like to be a school going teenager or young adult and what teenagers and young adults would like. Your answers will be used to improve care at schools for teenagers and youth. This project is being funded by the National Institutes of Health.*

*Before we begin talking with you about your experiences, we want to learn a little bit more about who you are by asking a few short questions.*

**Ask and complete demographic survey now.**

*Thank you. We are now ready to begin our longer discussion. During this conversation, I am interested in understanding all of your thoughts, experiences and opinions. I will ask you questions that you are free to answer in any way you wish. Your opinion is very important to us. You do not have to answer all the questions. If you want to stop the interview at any time, just tell me and we can stop.*

*There is no right or wrong answer to anything that I ask. If a question is unclear to you, please feel free to ask me to explain it.*

*The research team may take short sections of what you say and share them with non-study members but your name won’t appear anywhere. The research team will take information about your age, education, and HIV treatment experience and combine this information with all of the other teenagers and young adults doing these interviews.*

*I would like to record the interview so I don’t miss anything that you say. I will not include your full name on any documents or in the recording. Your answers will be kept confidential. The only time when we would break confidentiality is if you are under 18 and you are being abused.*

*Is it okay if I record our discussion? [Wait for the participant to give verbal consent to recording]*

*I am turning on the recorder now.*

**Before beginning interview questions, please read the following script for the recording:**

*Today is [day of week], [month, day, year] and it is now [time of day]. I am speaking with participant [participant ID].*

*Can you confirm for me again, by stating yes or no, that you are willing to participate in the conversation today?*

**You are now ready to begin asking the questions outlined below.**

**Semi-Structured Interview Questions**

*I would like to ask you some questions to learn more about your experiences with HIV care in school.*

**Question 1:** When you, or other adolescents who are living with HIV, are at school and feel sick, what do you/they do?

Where do you go for care? Who do you tell and what do they usually do?

- - Do you attend a different clinic than your regular HIV clinic to get your care? Why?
  - What is the process to ask for permission to go to clinic?
    - What information does the school need to give permission?
    - How long do you have to wait to get permission?
    - What if you have exams?

How is what you do when you are at school different than what you might do if you felt sick and you are at home? How is it the same?

Could you give an example of a time you needed medical care in school and got good support? What was done? How did that make you feel?

Could you give an example of a time you needed medical care in school and got poor support? What was done? How did that make you feel?

What could the school do to make the experience of attending clinic while in school better?

**Question 2:** What’s it like taking medicine when you are in school?

**ASK for ALL**

- How does taking medicine fit into your school schedule?
- Have you experienced medicine side effects when in school? What did you do about it?

**ASK for BOARDING ONLY**

- How do you store your medicine when in school?
  - Is this ok with you? Why?
- How do you take your medicine when in school?
  - Is this ok with you? Why?

Does the school provide you clean water and food to support you to take your medicine?

Who, if anyone, supports you to take your medicine when in school?

- Have you noticed other students who take medicine in school?

**ASK for ALL**

- What could make taking medicine in school a better experience?
  - What could day schools do differently that would make taking medicine easier? What about boarding schools? Why or why not?
- Prompts for day schools: Allow us to come to school a few minutes later or leave earlier, provide clean water, have a selected teacher or staff we can talk to about challenges with medication, allow us to carry medicine to school
- Prompts for boarding schools: Allow us to keep HIV medicine, allocate a specific staff to keep medicine, have a selected teacher or staff we can talk to about challenges with medication, provide private lockable cabinets to keep medicine, stop drug
  - What could schools do differently?

**Question 3:** Do you attend a support group?

- Do you think the support group helps you? How? Does the support group help you with managing your HIV while you are at school? Why or why not?
- Would you be interested in a support group that helps you manage your HIV when you are in school? Would you like this to be separate from your regular support group (if any)? Why or why not?
  - How would you like the support group look like?
    - Prompts: ehealth support group (WhatsApp, text messages), general health talks in school on various diseases, health clubs for adolescents with any chronic illness,

**Question 4:** Have you told anyone that you have HIV? If so, why did you decide to tell that person? How did they react?

- Have you told anyone in school you have HIV? If yes, why did you decide to tell that person? How did they react?
- Do you think HIV positive students should disclose their HIV status to someone at school? Why or why not?
- What do you think schools can do to help you and other student feel more comfortable talking about HIV?

**Question 5:** As you know, when it comes to time for upper primary or secondary school, some students go to day schools and some students to boarding school.

- Were you involved in the decision to choose boarding/day school? If yes, why did you choose day/boarding school? If not, who made the decision?
- Were you concerned that it might be harder to manage your clinic visits and medicine if you were at a boarding school?
- How do you think medication storage when in school could influence your decision to attend boarding or day schools? Why?

How does your experience receiving medical care while in school influence what schools you attend? Do they make you want to go to boarding school or day school? Why?

How do you think what other people think about HIV in school would influence your decision to attend boarding or day school? Why?

*Let’s summarize some of the key points from our discussion. [Summarize] Is there anything else you would like to add?*

*Do you have any questions for me before we end our conversation today?*

*Thank you for taking the time to talk to us*
