# Supplementary material for: “We can tell a good teacher who cares, understands, and can be confidential about it”: youth and caregiver experiences with HIV disclosure to schools in Kenya
Source: Front Public Health. 2023 Jul 25;11:1172431. doi: 10.3389/fpubh.2023.1172431 (PMC10407401; doi:10.3389/fpubh.2023.1172431)
Supplement: Supplementary file 2 [file Table_2.DOCX]

| **TIMIZA Study** |
| --- |
| **Caregiver**  **Interview Guide** |

**Interviewer Instructions:** Copies of the informed consent form should be provided to the participant and read aloud for the benefit of those who cannot read. Participants should be provided an opportunity to ask any questions. Verbal agreement to participate should be taped.

The following set of questions is a guide. Try to ask all the questions below in the order given, but it is more important to maintain the flow of discussion. Suggested probes have been included.

**Welcome and Introductions**

**Before turning on the recorder, start with the following introductory script:**

*Hi, my name is ____________. Thank you for agreeing to talk to me today. As mentioned earlier, I am a researcher and I am trying to learn more about how schools support HIV care for teenagers and young adults. Doctors and researchers have special knowledge about health and illness, but you are the expert on what it is like to be a caregiver of a school going teenager or young adult and what teenagers and young adults would like. Your answers will be used to improve care at schools for teenagers and youth. This project is being funded by the National Institutes of Health.*

*Before we begin talking with you about your experiences, we want to learn a little bit more about who you are by asking a few short questions.*

**Ask and complete demographic survey now.**

*Thank you. We are now ready to begin our longer discussion. During this conversation, I am interested in understanding all of your thoughts, experiences and opinions. I will ask you questions that you are free to answer in any way you wish. Your opinion is very important to us. You do not have to answer all the questions. If you want to stop the interview at any time, just tell me and we can stop.*

*There is no right or wrong answer to anything that I ask. If a question is unclear to you, please feel free to ask me to explain it.*

*The research team may take short sections of what you say and share them with non-study members. The research team will take information about your age, education, and caregiving experience and combine this information with all of the other parents and guardians doing this interview.*

*I would like to record the interview so I don’t miss anything that you say. I will not include your name on any documents or in the recording. Your answers will be kept confidential.*

*Is it okay if I record our discussion? [Wait for the participant to give verbal consent to recording]*

*I am turning on the recorder now.*

**Before beginning interview questions, please read the following script for the recording:**

*Today is [day of week], [month, day, year] and it is now [time of day]. I am speaking with participant [participant ID].*

*Can you confirm for me again, by stating yes or no, that you are willing to participate in the conversation today?*

**You are now ready to begin asking the questions outlined below.**

**Semi-Structured Interview Questions**

*The following questions will ask you about your experiences caring for a school going HIV-infected child or teenager. When answering these questions, think about yourself and your teenager’s experiences, and also about other teenagers and young adults with HIV.*

**Question 1:** What kind of things do you talk about to your teenager regarding school?

*Prompts: How to stay healthy, sexual education, maintaining good grades*

- Where do you get the information you share with your teenager?
  - Prompts: Lived experience, media, other mothers, church, support groups
- What kind of support do you think caregivers of teenagers need to talk to their teenagers?
  - Prompts: parenting classes, parenting material, support groups. Why
- What other topics do you talk about only to your teenager who is living with HIV?
  - Prompts: Medication use, what to do when unwell, HIV disclosure, stigma.
- Where do you get information to talk to your teenager who is living with HIV?
  - Prompts: Lived experience, media, other mothers, church, support groups
- What kind of support do you think caregivers of teenagers living with HIV need to talk to their teenagers?
  - Prompts: parenting classes, parenting material, support groups. Why?
- Could you give an example of a time a talk with your teenager went well? What material did you use as you talked to them? Did you feel you needed more support?
- Could you give an example of a time a talk with your teenager did not go well? What material did you use as you talked to them? Did you feel you needed more support?

**Question 2**: When your teenager is in school, what do they do if they are unwell?

- Who supports your teenager when he/she is unwell in school?
- Could you give an example of a time your teenager told you they had a good experience in school when they were unwell?
- Could you give an example of a time your teenager told you they had a bad experience in school when they were unwell?
- How can the school improve this process to make it better for your teenager?

**Question 3:** When your teenager is in school, do they ever have to attend the HIV clinic?

- What processes or actions do you as a caregiver have to do with the school or someone at the school to make sure your teenager attends clinic?
- How do you feel this process or actions are working for your teenager?
- What could the school do to make this process or actions better?

**Question 4:** What has your teenager told you about taking medicines when in school?

ASK FOR BOARDING ONLY

- How does the school require medicine to be stored and used?
- What do you think about this school requirement?
- Does the school provide clean water and food to support your teenager to take medicine?
- Could you give an example of a time your teenager talked to you about a good experience taking medicine in school.
- Could you give an example of a time your teenager talked to you about a bad experience taking medicine in school.

ASK FOR ALL

- What kind of support do you think your teenager and other teenagers living with HIV need to take medicine when in day school? What about boarding school? Why or why not?
  - Prompts for day school
    - Have selected teachers to talk about medication experiences as needed, allow them to come a little late or leave school early
  - Prompts for boarding school
    - Someone to support them. Who would this person be?
    - Flexibility in where to store medicine. What options would work?
    - Reminders: alarm clocks
    - Alternative packaging: medicine boxes

**Question 5:** Do you attend a support group?

- Does the support group help caregivers of teenagers living with HIV on school issues? How?
- Would you be interested in a support group of caregivers of teenagers living with HIV to support school related issues? Why or why not?
  - Would you like this to be separate from your regular group? Why or why not?

**Question 6:** Have you disclosed your teenager’s HIV status to anyone in school? If so, why did you decide to tell

this person? How did they react?

- Did you talk to your teenager before disclosing their HIV status? What did your teenager think? How did they react?
- Do you think caregivers of teenagers should disclose the teenager’s HIV status to someone in school? Why or why not?
- Apart from disclosing your teenagers HIV status, did you talk to anyone at the school about your teenager’s need to take medicine or to miss class sessions to attend clinic? If yes, how did that go?

**Question 7:** As you know, when it comes to time for upper primary or secondary school, some students go to day schools and some students to boarding school.

- Who made the decision for your teenager to go to boarding or day school? If you did, why did you choose boarding or day school? If you did not, who made the decision?
- Were you concerned that your teenager would stop taking their medicine if they were in boarding school? Why? Why not?
- Were you concerned that your teenager may be discriminated because of their HIV status? Why? Why not?

*Let’s summarize some of the key points from our discussion. [Summarize] Is there anything else you would like to add?*

*Do you have any questions for me before we end our conversation today?*

*Thank you for taking the time to talk to us!*
