# Supplementary material for: “We can tell a good teacher who cares, understands, and can be confidential about it”: youth and caregiver experiences with HIV disclosure to schools in Kenya
Source: Front Public Health. 2023 Jul 25;11:1172431. doi: 10.3389/fpubh.2023.1172431 (PMC10407401; doi:10.3389/fpubh.2023.1172431)
Supplement: Supplementary file 3 [file Table_3.docx]

Table 3: Additional quotes on disclosure experiences and perceptions

| **Supplementary table: Additional quotes** | | |
| --- | --- | --- |
| **Theme** | **Quote** | **Participant** |
| Disclosure is beneficial | *Once you share with them about your status and that you are taking drugs whenever you go ask for permission to go to the hospital, he/she accept it with ease* | 15-year-old female, day school |
|  | *We have a school nurse who knows my status so whenever I feel sick, I just go to her for medication or permission but if it gets worse, she refers me to this hospital* | 19-year-old female, boarding school |
|  | *Disclosure should be applicable in boarding schools where students miss the support they get from their parents and therefore should get someone whom they trust to share with their status and be able to get support whenever they need it.* | 14-year-old female, transitioning to secondary school |
| Disclosure is complicated/Stressful | *disclosing to one of the school staffs whom you trust to be supporting you to take the medicine … and before you disclose, you will have spent some time with him* | 18-year-old female, switched from day to boarding school |
|  | *Because people will start viewing you differently and some will look down upon you, some will even isolate themselves from you.* | 18-year-old female, boarding school |
|  | *Do you think HIV positive students should disclose to someone at school? Respondent: No, because of stigmatization* | 19-year-old female, boarding school |
|  | *Before a parent discloses, they need to be extremely sure of who they are talking to and not to disclose to just anyone. I think my daughter confided in the teacher because she trusted her because of how they used to relate, she even went to visit her teacher for a week after finishing the exams.* | Mother of 16-year-old female transitioning to secondary school |
| Positive post disclosure experiences | *He (the teacher) responded well and was impressed because my daughter is a good pupil who interacts well with others and has been healthy all through. I told him I have kept my daughter close and helped her through the journey.* | Mother of 15-year-old in day school |
|  | *I had to disclose and thankfully the teacher seemed to understand.* | Mother of 16-year-old transitioning to secondary school |
|  | *Even when I disclosed to her (school principal), I did not find any issue with her the way you find others going ahead and asking parents a lot of questions. I have never had any problem from my daughter.* | Mother of 19-year-old in boarding school |
| Mistrust of school staff a barrier | ***Interviewer:*** *Do you think HIV positive students should disclose their status to anyone in school?*  ***Respondent:*** *Yes, but to that one person whom you truly trust.* | 16-year-old female, day school |
|  | ***Interviewer:*** *Do you think positive students should disclose their status to someone at school?*  ***Respondent:*** *No, because this would be like revealing your weakest point to someone and you just never know when they will strike and hit you hard to the lowest level* | 18-year-old male, day school |
|  |  |  |
